# Migration corridors of adult Golden Eagles originating in northwestern North America

**DOI:** 10.1371/journal.pone.0205204

**Published:** 2018-11-21

**Authors:** Bryan E. Bedrosian, Robert Domenech, Adam Shreading, Matthew M. Hayes, Travis L. Booms, Christopher R. Barger

**Affiliations:** 1 Teton Raptor Center, Wilson, WY, United States of America; 2 Raptor View Research Institute, Missoula, MT, United States of America; 3 Lone Pine Analytics, Laramie, WY, United States of America; 4 Alaska Department of Fish and Game, Fairbanks, AK, United States of America; University of Alberta, CANADA

## Abstract

There has been increasing concern for Golden Eagle (*Aquila chrysaetos*) populations in North America due to current and future projections of mortality risk and habitat loss from anthropogenic sources. Identification of high-use movement corridors and bottlenecks for the migratory portion of the eagle population in western North America is an important first step to help habitat conservation and management efforts to reduce the risk of eagle mortality. We used dynamic Brownian Bridge movement models to estimate utilization distributions of adult eagles migrating across the western North America and identified high-use areas by calculating the overlap of individuals on population and regional levels. On a population level, the Rocky Mountain Front from east-central British Columbia to central Montana and southwestern Yukon encompassed the most used migration corridors with our study extent for both spring and fall. Regional analysis on a 100 x 200 km scale revealed additional moderate and high-level use corridors in the central British Columbia plateaus. Eagles were more dispersed in the spring until their routes converged in southern Alberta. High-use fall corridors extended farther south into central Wyoming. Knowledge of these high-use areas can aid in conservation and site planning to help maintain and enhance migratory Golden Eagle populations in western North America.

## Introduction

Conservation and management of raptors requires knowledge of ecology and demographics within the breeding, wintering, and migratory periods across life stages [1, 2]. For long-lived raptors occupying large landscapes, such as the Golden Eagle (*Aquila chrysaetos*), a thorough understanding of these parameters across all life stages can be extremely difficult to achieve. An increasing amount of attention has been paid to the management of Golden Eagles in North America due to apparent population declines [2–5] and because of the juxtaposition of development and protections afforded by the Migratory Bird Treaty Act (United States and Canada), the Bald and Golden Eagle Protection Act (United States) and the Species at Risk Act (Canada). Though recent data suggest that populations in the western United States are currently stable [6, 7], Golden Eagles still are considered at risk due to low reproductive potential in combination with habitat loss and increasing risks of direct fatality [8–12].

The expansion of industrial-scale wind power generation across North America, which can lead to direct fatalities of Golden Eagles, highlights the need to identify important eagle use areas. Many areas suitable for wind development overlap Golden Eagle habitats [9], but the degree to which those potential development areas overlap migration routes remains unknown for most regions in western North America. Similarly, increases in other development, such as oil and gas extraction or solar energy harvest, can lead to habitat fragmentation and increase direct mortality risk through the addition of power lines and roads.

Most studies of Golden Eagle habitat and space use have focused solely on the breeding season in the western United States (e.g., [10, 13–16]). Information from winter [13, 17] and during migration [17, 18] is more limited but also important for Golden Eagle conservation due to differences in habitat selection and mortality [2]. Mapping high-use Golden Eagle migration corridors is a necessary step to help identify regions of potential conservation importance [19].

There are several conventional methods to estimate animal space use from GPS derived location data from marked individuals, most notably kernel density estimators [20]. For Golden Eagles, kernel estimators have been used to define the relative frequency of occurrence of an individual or population in time and space (i.e., utilization distributions; [15, 16, 21–23]). However, kernel derived utilization distributions do not account for the temporal structure of animal location data and perform poorly for actively migrating animals [24].

Both Brownian bridge and subsequent dynamic Brownian bridge, movement models (dBBMM) have been used to map and prioritize migration pathways. These methods benefit from fewer assumptions than predictive models of movements/selection because routes are generally limited in geographic scope and used by many individuals within the population [25, 26]. To estimate utilization distributions for individuals that are actively migrating, the dBBMM improves upon previous methods by incorporating the distance and time between successive locations, location error, and a dynamic Brownian motion variance parameter based on an animal’s speed and direction [24, 26], allowing for a more accurate representation and separation of directed movements.

Sawyer et al. [25] first utilized Brownian bridge models to describe migration routes and population-level migration corridors for mule deer (*Odocoileus hemionus*). For avian migrants, Brownian bridge models have been used to describe migration of geese across Asia to help inform disease transmission routes [27] and Osprey (*Pandeon halieatus*) migration corridors across the United States [28]. Palm et al. [29] used dBBMMs to map waterfowl migration flyways in Asia. Mojica et al. [30] used dBBMMs to create a population-level utilization distribution for Bald Eagles in the eastern United States as a tool for evaluating wind energy development and other potential hazards to eagles.

Here, our goal was to identify migration corridors of adult Golden Eagles at the continental-scale in western North America using dBBMMs. We also sought to create a method by which we could assess regional-scale migration pathways using a quantitative approach based on sample size within our study extent. Our objective was to avoid biases associated with varying sample sizes across the study area and different eagle capture locations/seasons. The purpose of this study was to highlight high-use corridors and bottlenecks as a first step to helping inform management and planning decisions as they relate to migratory Golden Eagles in the western United States and Canada.

## Materials and methods

**Fig 1:**
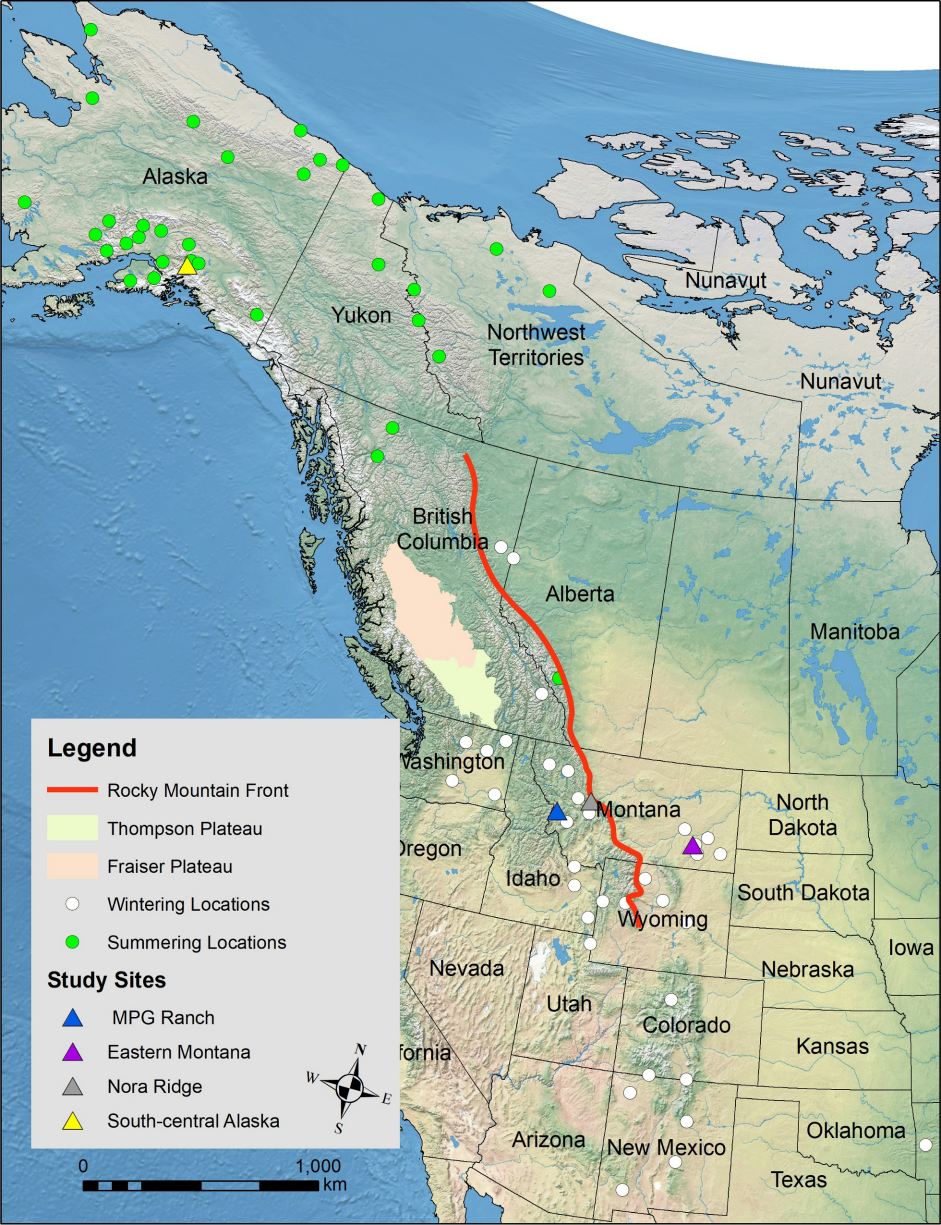
Generalized locations of study sites, geographic features referenced in text, and generalized summering/wintering locations of eagles used in the analysis.

Adult Golden Eagles were captured within six study areas as part of different but concurrent studies. We captured 16 over-wintering eagles within the “MPG Ranch study area,” which occurs within the Bitterroot Valley near Florence, MT (Fig 1). We tagged 16 actively migrating eagles at the “Nora Ridge” study site during September and October on the Continental Divide of the Rocky Mountains near Lincoln, Montana. Twenty-six eagles were tagged in the “Alaska” study area while they were on northward (i.e., spring) migration through southcentral Alaska. Finally, we captured five overwintering eagles across the Great Plains in Montana (“Eastern MT” study area). Additional data from two eagles were provided by the USFWS from the “4-Corners study” being conducted in the southwest US, and data from one eagle tagged in southeastern Wyoming were provided by the “FWS-Region 6 study.” Eagles from MPG Ranch, Eastern MT, Alaska, and 4-Corners were captured using net launchers (Trapping Innovations, LLC, Jackson, WY or Coda Enterprises, Mesa, AZ) baited with carrion. Eagles from the Nora Ridge study were captured using bow-nets with Rock Doves (*Columbia livia*) as bait. One eagle from the 4-Corners study was struck by a vehicle, rehabilitated, and released with a transmitter, and the eagle for the FWS-Region 6 study was captured using a carrion baited leg-hold trap. Methodologies used in this study were approved by Montana’s and Alaska’s Animal Care and Use Committees and conform to the Guidelines to the Use of Wild Birds in Research [31].

All eagles were fitted with either 45g or 70g solar Argos GPS transmitters (Microwave Telemetry, Inc, Columbia, MD) using a cross-chest harness of Teflon ribbon. Age was determined based on plumage [32]. Transmitters were programmed to gather 10–15 daily GPS locations during daylight hours. All data were collated, formatted, and processed through Movebank (www.movebank.org) and downloaded for analysis.

We examined data from all 64 eagles, which included 53 spring and 54 fall migration routes from 2011–2016. We visually inspected for and removed all obvious outliers (e.g., > 200km movement in one hour in a random direction from the directed movement) and any duplicate records using ArcGIS 10.5 (ESRI, Redlands, CA). We defined migratory movements as directed, continuous movements north or south >100km [30, 33] and extracted the first full fall and spring migration for all individuals. We removed any migrations that exhibited missing data for >48 h due to incomplete solar charging.

We only included one seasonal migration route per individual to eliminate pseudoreplication. All migration routes were from adult Golden Eagles (> 5-years-old at the time of migration). Several individuals were captured as sub-adults, but we only used data collected after the eagle reached breeding age. We did not differentiate among birds of different breeding status because most individuals were not visually confirmed as nesting. After applying these criteria to the data, we included 44 spring and 40 fall migration routes in the analyses. Thirty-two eagles contributed data to both spring and fall, while 20 additional eagles provided data for one season.

We transformed the location data into Alber’s equal area projection for all analyses. We used the MOVE package [34] in Program R [35] to calculate dynamic dBBMM utilization distribution for each individual migration route. We set the dBBMM parameters to the default margin of 13 and window size default of 31, with a location error of 150 m and a raster cell size of 2.5km. We visually assessed the fit of the default values to ensure they captured ca. one day of eagle movement and the variation in the data. We used 99% utilization distribution contours for each eagle/season and reclassified each so that 1 = used and 0 = unused surfaces.

We summed all reclassified utilization distributions by season to create population level UDs for spring and fall [25]. Mojica et al. [30] created a population level utilization distribution for Bald Eagles using a mean value for each cell, but this assumes equal probability of use by eagles across the entire spatial extent. We captured eagles at various locations during different seasons across our study area, so we could not make this assumption with our dataset. As a result, we created four regional-level population utilization distributions for both spring and fall: 25 x 50, 50 x 100, 100 x 200, and 200 x 400 km scales (north/south x east/west). We used focal statistics to calculate the maximum number of individuals within each window size and divided the summed UD for each 2.5 km^2^ cell by that total.

Sawyer et al. [25] used a threshold of 10% sample overlap as an intuitive metric to prioritize migration routes of ungulates. They further defined population-level corridors as low, moderate-low, moderate-high, and high use by binning the overlapping utilization distributions in 25% quartiles. We adopted these criterion to define the spatial extent of our analysis and conclusions as the areas in which > ca. 10% of our sample utilization distributions overlap (n = 4 individuals each season). We eliminated areas with <10% of the sample utilization distributions overlapping, which then defined our spatial extent. Within this extent, we visualized areas within the regional migration corridors as moderate-use and high-use based where 50–75% and > 75% of the sample overlapped within the moving window. Eliminating the use areas in which < 50% of the sample overlapped provided a conservative estimate of use corridors.

## Results and discussion

**Fig 2:**
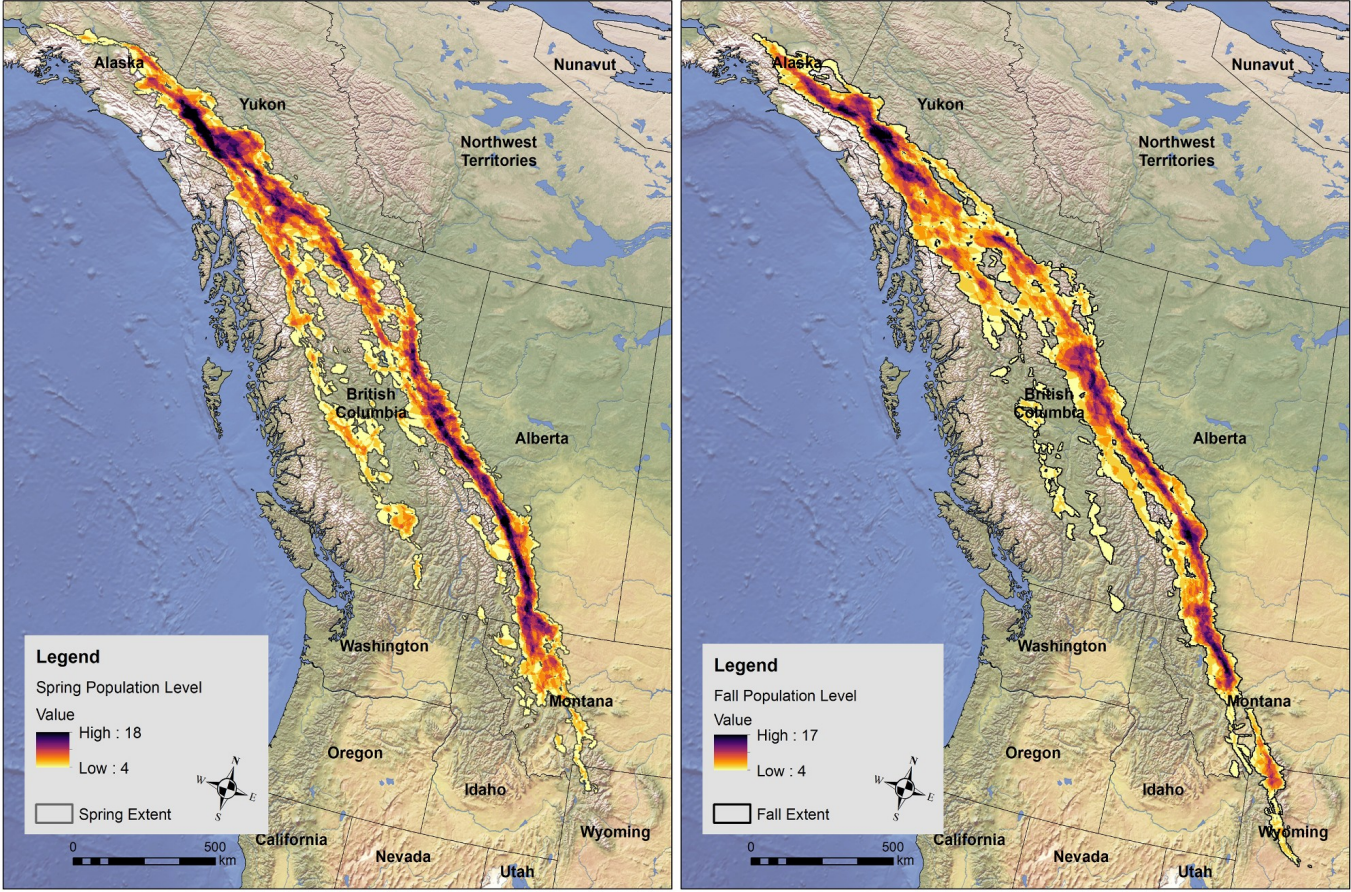
Population-level migration corridors of adult Golden Eagles during spring (left) and fall (right) migrations (2011–2016). Values within the spatial extent represent the number of overlapping individual eagle utilization distributions within each 2.5 km^2^ cell. The spatial extent was determined by a minimum of 10% of the sample population overlapping.

We identified Golden Eagle population level migration corridors for the spring and fall using the summation of all individual UDs [25] in both spring and fall (Fig 2). Using the population level utilization distribution summation, a maximum of 41% of our sample’s utilization distributions overlapped during the in the spring (n = 18) and 43% in the fall (n = 17). During the spring, eagles were more dispersed within the conterminous United States until they entered Canada where utilization distributions converged. Spring routes were slightly east on the Rocky Mountain Front and west on the interior route as compared to the fall. The clearest population level migration corridor in the fall was concentrated in southern Alaska through southwest Yukon then dispersed until north-central British Columbia where it became concentrated again along the Rocky Mountain Front from northern British Columbia into central Montana. Some individuals migrated farther west in central British Columbia in the Fraser and Thompson Plateaus (Level III Ecoregions [36]). The importance of the Rocky Mountain Front was similar in the spring, but routes were more concentrated within the western path.

**Fig 3:**
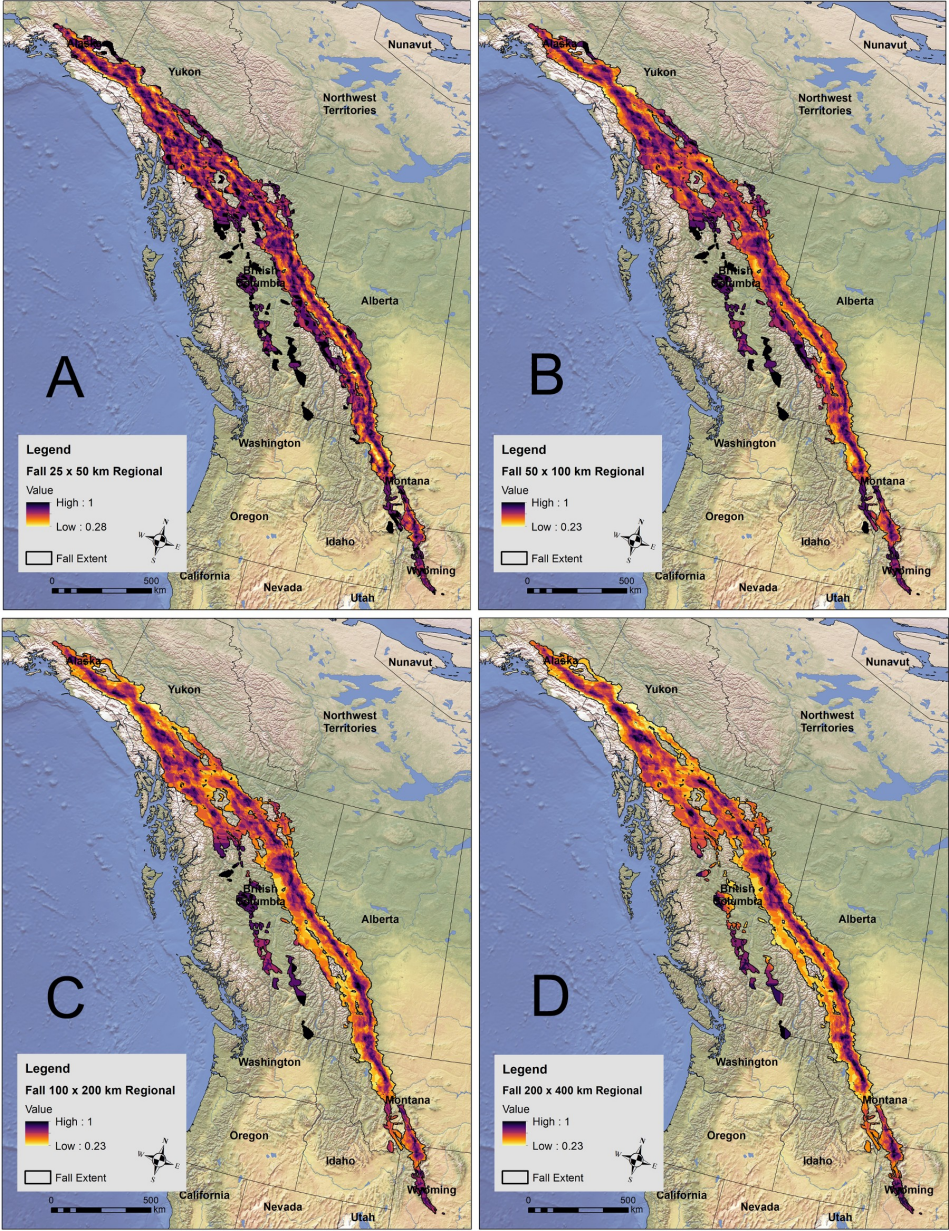
Regional-level Golden Eagle fall migration corridors defined by the proportion of eagles located within a moving window that used the particular 2.5 km^2^ center cell. Moving window sizes were 25 x 50, 50 x 100, 100 x 200 and 200 x 400 km (A-D, respectively).

The regional analyses highlight the importance of the Rocky Mountain Front for both spring and fall. We found that a moving window of 25 x 50 km was too small and over-estimated high-use routes in many areas due to small sample sizes (Fig 3). There was little difference between the remaining window sizes, but we concluded that the 100 x 200 km window size was the most appropriate scale to define population-level routes since the width of the 50% UD overlap was approximately 100 km. There was very little difference between the 100 x 200 km and 200 x 400 km scale (Fig 3), indicating that the 200 x 400 km scale was likely too large to detect any selection at that scale.

**Fig 4:**
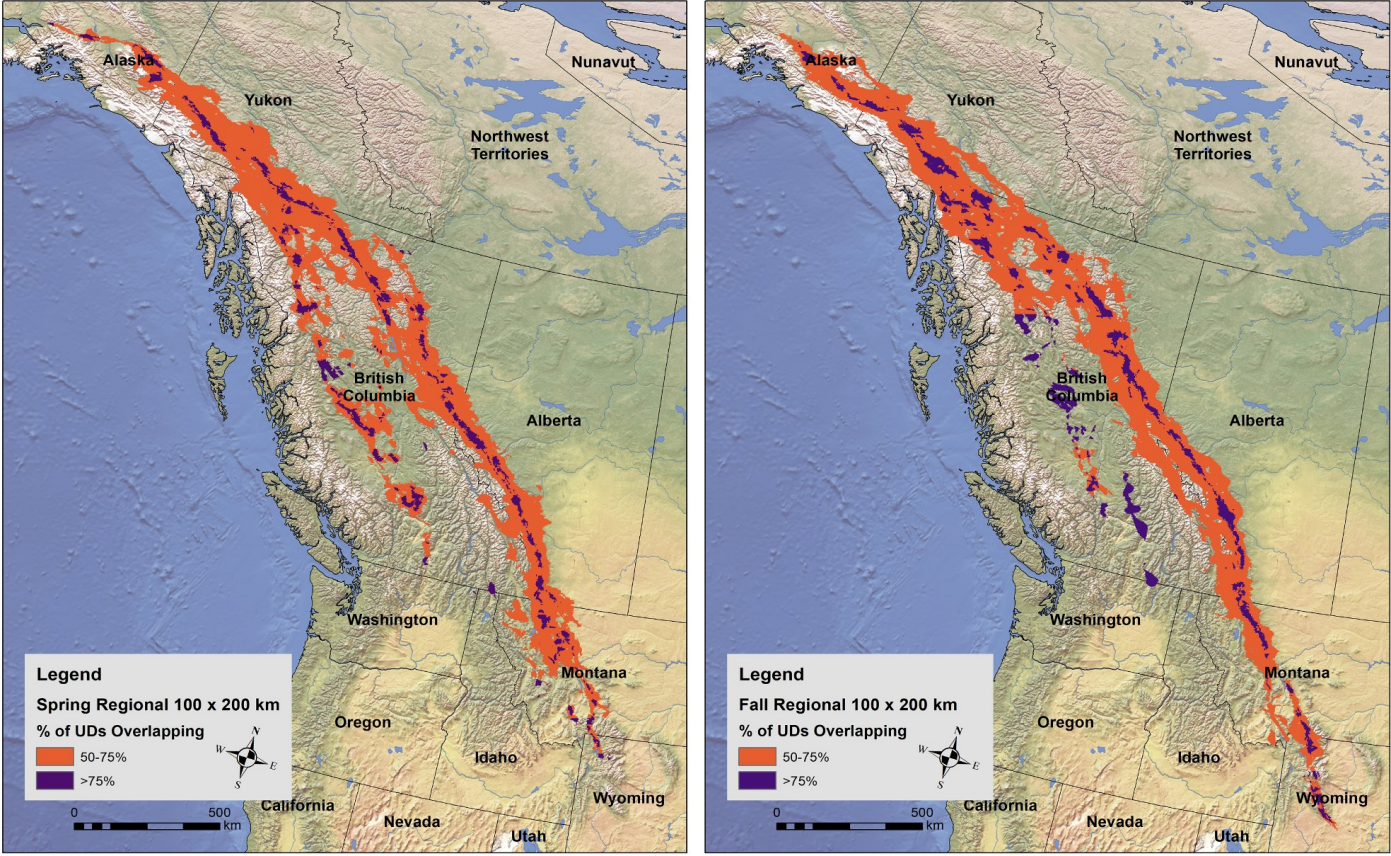
Final estimations of regional moderate- and high-use Golden Eagle spring (left) and fall (right) migration corridors in western North America using the proportion of eagles using a particular 2.5 km^2^ cell within a 100 x 200 km moving window. Moderate- and high-use corridors were determined by 50–75% and >75% eagle overlap within the moving window, respectively.

There was one major divergence/convergence area in both spring and fall (Fig 4). It appears that two distinct routes diverge in the fall and converge in the spring in northern British Columbia, with the western routes ending on wintering areas in the eastern British Columbia/Washington regions and the eastern route ending east of Idaho, Utah and Arizona. Spring regional routes were dispersed until they converged in southern Alberta, while eagles in the fall traveled along a concentrated path well into Wyoming.

## Discussion

Our analysis takes an initial step toward identifying high-use spring and fall migration corridors of adult Golden Eagles in western NA. Identification of specific high-use areas and migration bottlenecks can help inform conservation strategies, initial development site planning, and provide a base for future studies. Use of these data to help inform siting of new developments, such as energy extraction, outside identified high-use migration areas may reduce eagle take and compensatory mitigation needs from these areas. Prioritizing conservation efforts within high-use areas may help direct limited resources and maximize gains.

Based on the general knowledge of eagle migration, we feel that our sample reasonably represents the migratory population of Golden Eagles in western NA, particularly the corridors between 60° north (Yukon/Northwest Territories southern border) and 49° north (the United States border). Within this area, the high-use population-level migration corridor occurs along the eastern edge of the Rocky Mountains. One-third of our sample used this key pathway during both spring and fall migration (Fig 2).

Both population-level and regional interpretations of our results can provide valuable insights about adult Golden Eagle migration routes. At the population level, our data highlight an important migration corridor along the Rocky Mountain Front, with particular conservation implications across Alberta, northern Montana, and southwest Yukon. The population of migratory eagles in western North America occupies a wide breeding range across all of Alaska, Yukon and eastern Northwest Territories. Likewise, they over-winter across almost all of southwest Canada and the western conterminous United States.

At the northern and southern ends of the population level corridor we identified, eagles tend to disperse to widespread summering and wintering territories. As a result, fewer eagles utilize corridors at these extents and this dilutes the relative importance at the population-level corridor. However, high priority corridors still exist at smaller, regional scales (e.g., at the State or Territory-level). While a smaller portion of the overall migrating Golden Eagle population may use a particular region less frequently, there still may be a need to identify migration corridors for that portion for a specific subset of the population. Our regional analysis provides a tool for managers to assess relative importance of Golden Eagle migration routes within our study extent.

The majority of eagles within our sample wintered in Montana, Wyoming, Colorado and New Mexico. Several Alaskan summer residents wintered in southern British Columbia and Washington. The migration routes of these latter eagles were further west of all other eagles, along the Thompson and Fraser Plateaus. We identified this route by our methods, but the importance of this route may be understated due to relatively small sample sizes within this region. High-use migration routes for Golden Eagles surely exist beyond our study extent, and additional data may identify potential routes those routes.

Importantly, we did identify areas of high conservation priority for Golden Eagle migration. Lack of detection was a function of the geographic distribution of the studies included in this analysis, which did not include adequate samples of birds wintering south of Montana or breeding in northern Alaska, Yukon or Northwest Territories. Regardless, within our study extent, the data clearly help define moderate and high-use migration corridors for that area. Further data collection and collaborations are needed to more clearly identify key migration routes south of Montana, in other parts of Alaska, Yukon, and Northwest Territories, and the relative proportion of the western Golden Eagle migratory population that use those routes.

## Conclusions

Knowledge of these key migration routes has obvious conservation implications in light of increased industrial wind development across western North America and the accompanying potential hazards for Golden Eagles. Some of the best predicted wind resources and commercial wind development in the United States lie at the ecotone of the Rocky Mountains and the Great Plains [37], and there are clear conflicts with wind production and Golden Eagle survival (e.g., [9, 11, 12]). Similarly, southern Wyoming hosts some of the best wind potential in the United States [37], as well as the highest development potential areas [38], which may intersect high-use Golden Eagle migration corridors.

There are potential benefits to both Golden Eagle populations and development companies if our dataset and similar migration corridor mapping results are used in initial site planning. Avoidance of areas with a high risk for eagle mortality during migration may strengthen eagle take permit applications, reduce compensatory mitigation needs, and reduce liability for companies while simultaneously protecting migratory eagles.

The importance of migratory pathways of Golden Eagles in conservation planning often has been overlooked because of the large sample sizes and spatial extent needed to make inferences [19]. Our analysis is among the first attempts to objectively quantify the relative spatial importance of migration routes for adult Golden Eagles in western North America at both population and regional levels. Our focus on adult Golden Eagles also allows for the greatest conservation impact to migratory populations, as retaining and enhancing adult survival will have the largest impact on population levels [39]. Targeted conservation actions and minimization of mortality risks within these migration corridors will certainly benefit Golden Eagles in the Western Hemisphere.
